# Adherence to the Nordic Nutrition Recommendations in a Nordic population with metabolic syndrome: high salt consumption and low dietary fibre intake (The SYSDIET study)

**DOI:** 10.3402/fnr.v57i0.21391

**Published:** 2013-12-16

**Authors:** Svandis Erna Jonsdottir, Lea Brader, Ingibjorg Gunnarsdottir, Ola Kally Magnusdottir, Ursula Schwab, Marjukka Kolehmainen, Ulf Risérus, Karl-Heinz Herzig, Lieselotte Cloetens, Hannah Helgegren, Anna Johansson-Persson, Janne Hukkanen, Kaisa Poutanen, Matti Uusitupa, Kjeld Hermansen, Inga Thorsdottir

**Affiliations:** 1 Unit for Nutrition Research, Landspitali – The National University Hospital of Iceland and Faculty of Food Science and Nutrition, University of Iceland, Reykjavik, Iceland; 2 Department of Medicine and Endocrinology MEA, Aarhus University Hospital, Aarhus, Denmark; 3 School of Medicine, Institute of Public Health and Clinical Nutrition, University of Eastern Finland, Kuopio Campus, Kuopio, Finland; 4 Research Unit, Kuopio University Hospital, Kuopio, Finland; 5 Institute of Clinical Medicine, Internal Medicine, Kuopio University Hospital, Kuopio, Finland; 6 Department of Public Health and Caring Sciences, Clinical Nutrition and Metabolism, Uppsala University, Uppsala, Sweden; 7 Department of Physiology, Institute of Biomedicine and Biocenter of Oulu, University of Oulu, Oulu, Finland; 8 Department of Psychiatry, Kuopio University Hospital, Kuopio, Finland; 9 Biomedical Nutrition, Pure and Applied Biochemistry, Lund University, Lund, Sweden; 10 Department of Clinical Nutrition, Skåne University Hospital, Lund, Sweden; 11 Department of Internal Medicine, Institute of Clinical Medicine, University of Oulu, and Biocenter Oulu, Oulu, Finland; 12 Oulu University Hospital, Oulu, Finland; 13 VTT Technical Research Centre of Finland, Espoo, Finland

**Keywords:** diet records, dietary fibre, guideline adherence, metabolic syndrome, multicenter study, sodium, dietary

## Abstract

**Background:**

The Nordic countries collaborate in setting recommendations for intake of nutrients by publishing the Nordic Nutrition Recommendations (NNR). Studies exploring how well the Nordic population adheres to the NNR are limited and none are available for the metabolic syndrome (MetS) subgroup. Individuals with MetS are a large part of the adult Nordic population and their diet's nutritional quality is of great importance as it can affect the progression of MetS.

**Objective:**

To evaluate nutritional intake in a cohort of Nordic adults with MetS or MetS risk factors and their adherence to the NNR.

**Design:**

A multi-centre study was carried out in six centres in four Nordic countries (SYSDIET CoE). Participants (*n*=175) were 30–65 years of age, with BMI 27–38 kg/m^2^ and had at least two criteria for MetS. The NNR was used to evaluate the baseline nutrient intake calculated from the participants’ 4-day food diaries using national nutrient databases.

**Results:**

Less than 20% of participants consumed ≤10 E% from saturated fat as recommended in the NNR. Recommended intake (RI) of polyunsaturated fat was met by approximately one-third of participants. Only 20% of men and 26% of women met the RI of dietary fibre. Intake below the defined lower intake level of 2.5 µg/day for vitamin D was observed in nearly 20% of participants. The daily median intake of salt was 8.8 g for men and 6.7 g for women.

**Conclusion:**

Dietary quality of this Nordic population with Mets or MetS risk factors is unsatisfactory and characterised by high intakes of SFA and sodium and low intakes of PUFA and dietary fibre. Vitamin D intake was below RI level in a large part of the population. Authorities in the Nordic countries are encouraged to develop intervention programmes for high-risk groups.

Prevalence of overweight, obesity, and sedentary lifestyle has increased worldwide (1–3). Trends show declining rates for coronary heart disease (CHD) and stroke mortality, attributed at least partly to the reduction in cardiovascular risk factors in the population (3, 4). The incidence of type 2 diabetes (T2D) is increasing rapidly and is considered an epidemic (3).

Metabolic syndrome (MetS) is characterised by the presence of multiple metabolic risk factors for cardiovascular diseases (CVD) and T2D. In line with findings in T2D, MetS has a rising prevalence worldwide (2). The prevalence of MetS increases strongly with age, especially in women (5). This is alarming considering that the MetS markedly increases mortality, also in Nordic populations (6). Recent Nordic studies have shown MetS prevalence ranging from 9.2 to 64.4% in age- and sex-specific groups (5, 7, 8), with even more people having single risk factors for MetS (5). Contributing to the different prevalence reported in the studies may be different MetS criteria and different age–sex distribution. A recent comparison between four definitions of MetS showed higher prevalence with the definition by Alberti et al. (2) than other definitions (9). Many of the non-communicable disease determinants are related to unfavourable diet, low physical activity (PA), and associated risk factors (10–14) with T2D being largely preventable with the modification of risk factors (3).

The need for joint efforts in the European public health arena has been identified at many levels, ranging from recognition of the need for competent public health workforce (15, 16) to the necessity for the standardisation of dietary assessments (17). The need for collaboration to develop harmonised diet-related public health actions in Europe has also become apparent through the emerging numbers of multi-centred nutritional studies (17–21). The Nordic countries have for several decades collaborated in setting recommendations for intake of nutrients by publishing the Nordic Nutrition Recommendations (NNR) (22). Yet, studies exploring how well the Nordic population adheres to the NNR are limited. As individuals with or at risk of MetS are a large part of the adult Nordic population (5, 7, 8) the nutritional quality of their diets is of great importance and interest. Consequently, knowing the nutritional situation of the Nordic population before developing and implementing effective intervention programmes aiming to improve nutrition is of great significance. The knowledge of the present situation in the Nordic countries for this subpopulation of people with MetS and MetS risk factors can help tailor future prevention policies and dietary recommendations aimed at this vulnerable group. It is of importance to identify how well this Nordic subpopulation adheres to general nutrition recommendations (i.e. the NNR) aimed at the public to identify potential targets for actions.

The aim of the present study was to gain insight to and evaluate the nutritional intake in a cohort of Nordic adults with MetS or MetS risk factors and their adherence to the NNR. This is the first multi-centred Nordic study assessing the nutrient intake in a Nordic population with MetS or at high risk of MetS, and their adherence to the NNR. This study was conducted by the SYSDIET consortium (Systems biology in controlled dietary interventions and cohort studies) within the Nordic Centre of Excellence/Norden.

## Materials and methods

The SYSDIET intervention study was a randomised controlled multicentre study performed in six intervention centres distributed in Denmark (Aarhus), Finland (Kuopio and Oulu), Iceland (Reykjavik) and Sweden (Lund and Uppsala). Local ethical committees approved the SYSDIET study protocol, which followed the Helsinki declaration guidelines. Informed written consent was obtained from all participants.

## Study population

**Fig. 1:**
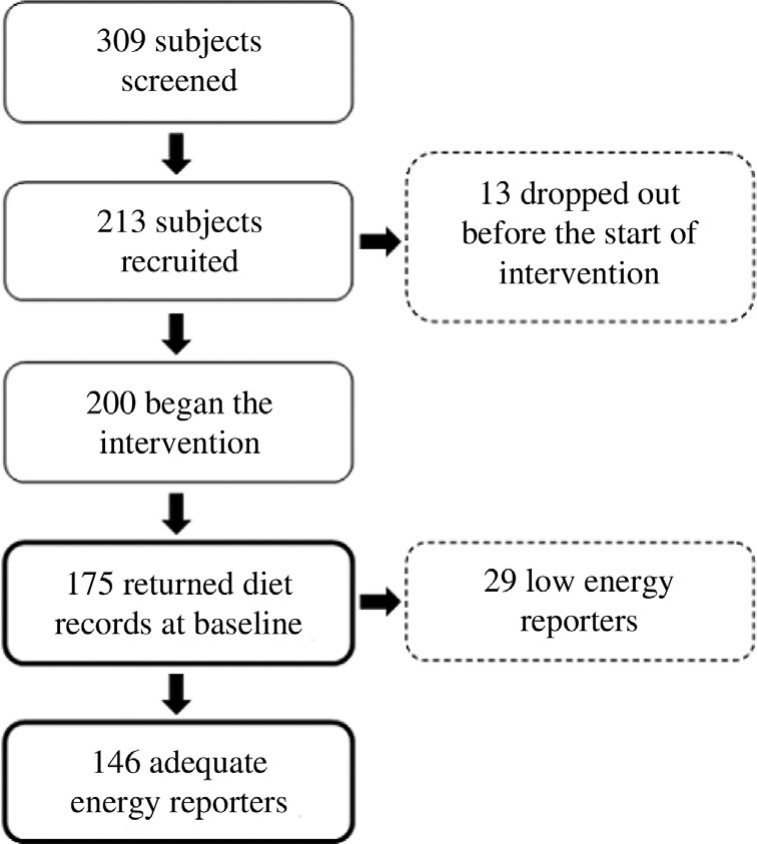
Schematic overview of the participant flow.

Participants were recruited primarily through advertisements in newspapers and at public places, and from previous clinical or epidemiological trials in the study centres. The inclusion criteria were 30–65 years of age, body mass index (BMI) 27–38 kg/m^2^ and at least two criteria for MetS. Definitions of MetS risk factors used in the present study where elevated waist circumference (≥94 cm for men and ≥80 cm for women), triglycerides concentrations ≥1.7 mmol/l, HDL <1.0 mmol/l in men and <1.3 mmol/l in women, elevated blood pressure (systolic blood pressure ≥130 and/or diastolic pressure ≥85 mm Hg) and fasting plasma glucose ≥5.6 mmol/l (2). Drug treatment for these MetS components was used as alternative indicators of the respective risk factors (2). The main exclusion criteria included diabetes and any chronic disease or condition which could hamper the dietary protocol being followed, BMI >38 kg/m^2^, fasting plasma glucose >7.0 mmol/l, fasting triglycerides >3.0 mmol/l, total cholesterol >6.5 mmol/l, and blood pressure >160/100 mmHg. Despite of the BMI criteria, few study participants with BMI between 38 and <40 kg/m^2^ were accepted due to high commitment to the trial. The intervention protocol, including the inclusion and exclusion criteria, has been described previously (23). Figure 1 illustrates the participant flow for the present study. Completed diet records were returned by 175 participants – 60 men and 115 women.

## Dietary assessment

Dietary assessment was conducted in the run-in period of the SYSDIET study, where participants were instructed to maintain their usual diet. Diet and nutrient intake were assessed by diet records where participants were instructed to record all food and drink consumed for four consecutive days, including one weekend day. Food intake was reported either as weight or portion size. Supplementation and plant stanol/sterol containing products intake if any, was to be discontinued at least 4 weeks prior to entering the study, with the exception that Icelandic participants could continue intake of vitamin D supplements providing they kept it unchanged. The vitamin D supplementation was not included in the nutrient calculations. Participants received both written and oral instructions on how to fill in the diet records and were provided with electronic household scales or valid standardised household measure information on how to give information about the amount of food consumed.

Local nutrient calculating programmes, supported by the national databases (Table 1) were used to estimate energy and nutrient intake. The nutrient databases are comparable, use EuroFIR definitions for chemicals (24), determine dietary fibres by the AOAC methods and define added sugar as refined or industrially manufactured sucrose and other sugars, eventually in the form of an ingredient in a food (25–28). The same energy conversion factors of energy-providing nutrients were used in all four databases: 4 kcal/g for protein and (available) carbohydrates, 2 kcal/g for dietary fibre, 9 kcal/g for fat, and 7 kcal/g for alcohol. Sodium was transformed to salt by multiplying by 2.54.

**Table 1:** Nutrient and food databases and nutrient calculation programmes used within countries

	Nutrient and food database	Nutrient calculation programme
Denmark	Danish National Food administration database	Master Dietist System, v. 1.235 (2007)
Finland	Fineli – the Finnish Food Composition Database	Diet 32 nutrient calculation software, v. 1.4.6.3, Aivo Finland Ltd, Turku, Finland
Iceland	Isgem – the Icelandic Food Composition Database	Icefood, v. 2.0 (2010)
Sweden	Livsmedelsdatabasen – the Swedish Food Database 2009	Dietist XP Software Package, v. 3.1 (2009)

The Nordic reference values (NRVs) for energy intake (EI) and recommended macronutrient intake for adults were used to evaluate the dietary intake. Intake of four vitamins (vitamins C, D, E, and folate) and four minerals (calcium, magnesium, potassium, and sodium) was estimated and compared to the recommended intake (RI) (22). These micronutrients were chosen by the SYSDIET management team and considered to be especially relevant for the Nordic diet. Furthermore, the Nordic nutrient databases were considered to give adequate and comparable information on these selected micronutrients. For nutrients with established estimates of average requirement (AR) (vitamins C, E, and folate), AR was used to assess the risk of inadequate intake. Proportion of the population not meeting the lower level of intake (LI) of micronutrients or exceeding the upper intake level (UL) was also assessed (22).

## Low-energy reporting

Height of participants was measured in a standing position in the morning to the nearest 0.5 cm. Body weight was recorded to one decimal place, using calibrated electronic scales while the subject wore light indoor clothing without shoes. Low-energy reporting was evaluated using the Goldberg cut-off 2 (29, 30), comparing the ratio of mean EI and calculated basal metabolic rate (BMR). Harris–Benedict (HB) equations (31) were used to estimate BMR. Ideal body weight (IBW) was calculated using the method proposed by Hamwi (32). If participants weighed ≤120% of their IBW, their actual weight was used in the HB calculations rather than their adjusted body weight (33). Adjusted body weight was used in BMR calculations for participants weighing >120% of their IBW, assuming that 25% of the excess weight was lean tissue (33). Age- and sex-specific mean physical activity level (PAL) values derived from doubly labelled water studies (34) and modified in Black et al. (35), were applied to participants in calculations as the SYSDIET questionnaire did not provide complete information on total PA. The Goldberg cut-off 2 (29, 30) calculated specifically for the current study on an individual level (36) was 1.13, resulting in the definition of low-energy reporters if mean EI:BMR <1.13 and adequate reporters if mean EI:BMR ≥1.13.

## Biochemical measurements and body composition

Biochemical measurements were all completed locally at each centre, except for fasting plasma insulin, which was analysed at the Aarhus University Hospital using the ELISA method. Automated clinical chemistry analysers and routine clinical chemistry methods were used to measure concentrations of fasting plasma glucose and fasting serum triglycerides, total cholesterol and HDL-cholesterol. Fasting LDL-cholesterol levels were calculated (37). Blood pressure was measured by trained personnel using automatic blood pressure devices. Where possible, blood pressure was measured on the right arm of participants after 10 min rest in a sitting position. The average blood pressure of two or three measurements was recorded with the accuracy of 1 mm Hg. Waist circumference was measured midway between the lower rib and iliac crest. Body composition was determined by bioelectrical impedance analysis. Staff at each centre was trained to perform the measurements according to the standard operational procedures agreed by all centres. Clinical and biochemical measurements have been previously described (23).

## The SYSDIET questionnaire on socio-demographic factors and lifestyle habits

Participants answered questionnaires on socio-demographic factors, health status, physical exercise, and other lifestyle habits. They were asked about age, marital status, education, profession, and their subjective health status (good, average, or poor). They were provided with a list of diseases and asked to mark (yes/no) if they had been diagnosed with them. They were also asked to provide detailed information about all regular medication. Participants were asked questions on PA at work, commuting and during leisure time. One PA question focused on how many times per week the participants performed fitness training for ≥30 min (≥4 times/week; 2–3 times/week; 1 time/week; 2–3 times/month; <2 times/month). These questions have been used previously to assess PA (38–41), especially in evaluating if PA is held constant among participants during an intervention study.

## Data pooling and analysis

All SYSDIET centres entered their data into Microsoft Office Excel 2007 (Windows) prior to pooling the data in a joint centralised database maintained by VTT Technical Research Centre of Finland. Data were exported from the database and imported to SPSS (Statistical Package for the Social Sciences) for Windows, version 20.0 (SPSS Inc., Chicago, IL) for statistical analyses.

Normality of variables was checked by visual inspection and by using the Kolmogorov–Smirnov test. Variables are described as means and standard deviations (SD) or medians and interquartile range (IQR).

Independent sample *t*-tests were used to compare anthropometric and biochemical data and nutrient intake between genders and adequate reporters versus low-energy reporters when variables were normally distributed, whereas Mann–Whitney's *U* test was applied if variables had non-normal distribution. Pearson's Chi-square test or Fisher's exact test (if expected frequencies <5), were used to test for differences in frequency distributions between genders, and also between low-energy reporting men/women versus adequately reporting men/women, in relation to marital status (cohabitation or not), education (university level vs. lower education), profession (manual labour vs. sedentary work), smoking habits (never smoked/ceased smoking vs. smokes regularly/occasionally), blood lipid-lowering medication, hypertension treatment or antidepressant medication (taking medication vs. not) and PA (participants exercising ≥4 times a week for ≥30 min vs. less PA). *P* <0.05 was regarded as significant in all analysis.

## Results

Characteristics of participants returning adequate diet records at baseline (*n*=146) are shown in Table 2. Of the 146 participants, all but one man had elevated waist circumference according to the IDF criteria (2). Additionally, 29% had elevated triglyceride, 33% had reduced HDL cholesterol, 57% had elevated blood pressure, and 61% had elevated fasting glucose. Furthermore, 52% were treated for hypertension, 23% were on blood lipid-lowering medication but none were treated for T2D as it was an exclusion criterion. When asked to rate their own health, 115 of the 146 participants responded, with 55% rating their health as good, 44% as average and one individual as poor.

**Table 2:** Characteristics of participants returning adequate diet records at baseline

Characteristics	Men (*n*=46)	Women (*n*=100)	*P*
Age (years)	55.0 (8.5)	55.0 (8.3)	0.985
Weight (kg)	99.9 (10.7)	85.9 (11.4)	<0.001
Waist circumference (cm)	109.9 (8.3)	101.0 (8.5)	<0.001
BMI (kg/m^2^)	31.1 (2.8)	31.9 (3.6)	0.130
Body fat (%)	31.6 (6.2)	43.0 (4.4)	<0.001
Systolic BP (mm Hg)	136 (15)	127 (15)	0.001
Diastolic BP (mm Hg)	84 (11)	80 (10)	0.053
Total cholesterol (mmol/l)	5.0 (0.9)	5.4 (0.9)	0.038
LDL (mmol/l)	3.1 (0.8)	3.3 (0.9)	0.379
HDL (mmol/l)	1.2 (0.3)	1.5 (0.4)	<0.001
Triglyceride (mmol/l)^a^	1.4 (0.8)	1.3 (1.0)	0.232
Glucose (mmol/l)	5.8 (0.7)	5.7 (0.6)	0.287
Insulin (pmol/l)^a^	58.0 (43.0)	50.0 (39.0)	0.203
Married/cohabitating (%)	82.5	69.7	0.126
University education (%)	25.8	13.0	0.106
Sedentary work (%)	88.4	96.7	0.110
Smokers^b^ (%)	10.9	10.0	1.000
Antidepressants%	9.8	10.6	1.000

Values are mean (SD) unless otherwise indicated.

BMI, body mass index; BP, blood pressure; LDL, low-density lipoprotein; HDL, high-density lipoprotein.

a Medians and interquartile range.

b Smoking regularly or occasionally.

The percentage of low-energy reporters was 17%, 23% for men and 13% women, respectively. Low EI reporting men had significantly higher (*P*=0.043) BMI than adequate intake reporters (33 kg/m^2^ vs. 31 kg/m^2^). Low EI reporting women reported more frequent PA than adequately reporting women, with 36% of low EI reporters vs. 4% of adequate EI reporters exercising ≥4 times a week for ≥30 min (*P=*0.001). A significant difference (*P*=0.038) was seen for education level, as a higher percentage of low-energy reporting women (39%) had university education than adequately reporting women (13%). Furthermore, there was a trend of antidepressants intake in low EI reporting women (29%) compared to women with adequate EI reporting (11%) (*P*=0.083). Other characteristics listed for the adequate reporters in Table 2 and medication intake (blood lipid-lowering and hypertension treatment) were not significantly different between low-energy vs. adequate EI reporters.

In Tables 3–5, energy and nutrient intake is presented for adequate energy reporters. Table 3 presents daily intake of energy and macronutrients, the RI of macronutrients according to NNR (22) and the proportion of participants with nutrient intake in line with the recommendations. Contribution of saturated fatty acids (SFA) to total EI was, on average, higher than the RI, and ≤20% of participants consumed <10 E% from SFA (Table 3). The RI of 5–10 E% PUFA was only met by 35% of women and 30% of men (Table 3).

**Table 3:** Baseline energy and macronutrient intake per day of adequate energy reporters and adherence to NRV

		Men (*n*=46)		Women (*n*=100)		
				
Energy and macronutrients	NRV	Mean (SD)	Within NRV (%)	Mean (SD)	Within NRV (%)	*P*
Energy (kcal)		2438 (392)		1997 (397)		<0.001
Total fat (E%)	25–35	32.7 (6.8)	48	34.1 (5.8)	47	0.209
SFA (E%)	≤10	12.8 (3.4)	20	13.2 (3.3)	14	0.516
MUFA (E%)	10–15	11.0 (2.7)	48	11.5 (2.6)	59	0.214
PUFA (E%)^a^	5–10	4.2 (1.7)	30	4.7 (2.2)	35	0.064
Total carbohydrates (E%)	50–60	43.7 (6.4)	24	46.0 (6.3)	30	0.046
Sucrose (E%)	≤10	6.7 (3.5)	80	8.3 (3.5)	74	0.013
Dietary fibre (g/d)^a^	25–35	22.0 (8.3)	20	21.0 (8.8)	26	0.794
Dietary fibre (g/MJ)^a^	≥3	2.1 (0.8)	13	2.6 (1.0)	30	<0.001
Protein (E%)	10–20	17.3 (2.7)	85	16.3 (2.5)	93	0.026
Protein (g/kg body weight)^a^	0.8	1.0 (0.3)	98	0.9 (0.3)	75	0.007
Alcohol (E%)^a^	≤5	3.5 (7.7)	65	0.4 (4.7)	76	0.002
Alcohol (g/d)^a^^,^^b^	≤20/ ≤ 10	12.0 (24.2)	70	1.3 (13.8)	72	0.001

*P* values refer to the difference in energy and macronutrient intake between genders.

NRV, Nordic reference values.

a Medians and interquartile range.

b The higher NRV for alcohol (g) refers to men, the lower to women.

**Table 4:** Baseline micronutrient intake per day of adequate energy reporters and adherence to NRV

				Men (*n*=46)				Women (*n*=100)				
						
Micronutrients	RI	AR	LI	Median (IQR)	Below/above^a^ RI%	Below AR%	Below LI%	Median (IQR)	Below/above^a^ RI%	Below AR%	Below LI%	*P*
Vitamin C (mg)	75	60/50^b^	10	89.0 (71.0)	35	24	2	104.0 (78.8)	28	11	0	0.159
Vitamin D (µg)	7.5/10^c^	–	2.5^d^	5.8 (6.7)	65	–	17	5.0 (5.8)	74	–	19	0.262
Vitamin E (mg)	10/8^b^^,^^e^	6/5^b^^,^^e^	4/3^b^^,^^e^	8.8 (4.5)	65	15	0	8.8 (4.4)	40	5	0	0.898
Folate (µg)	300/400^f^	200	100	289.5 (161.5)	52	13	0	265.5 (110.3)	71	17	0	0.272
Calcium (mg)	800	–	400	1061.7 (659.3)	24	–	0	860.5 (430.1)	42	–	5	0.001
Magnesium (mg)	350/280^b^	–	–	385.0 (209.5)	39	–	–	325.8 (125.2)	26	–	–	0.001
Potassium (mg)	3500/3100^b^	–	1600	3588.0 (1814.7)	48	–	0	3221.5 (1298.7)	45	–	0	0.003
Sodium (mg)	<2800/ < 2400^b^	–	575	3460.2 (1155.4)	83^a^	–	0	2624.5 (947.5)	65^a^	–	0	<0.001

Reference values not defined in NNR (22).

*P* values refer to the difference in micronutrient intake between genders.

NRV: Nordic reference values; RI: recommended intake; AR: average requirement; LI: lower level of intake; IQR: interquartile range.

a Percentage of participants above the recommendations for sodium.

b Reference values for men/women.

c The lower RI value for vitamin D applies to people 31–60 years old and the higher RI value applies to people 61–74 years old.

d The LI for vitamin D is primarily set for individuals >60 years of age, but percentage and numbers of all men and women below the LI are presented.

e α-Tocopherol equivalents (α-TE = 1 mg RRR-α-tocopherol).

f Women of reproductive age are recommended an intake of 400 µg folate/day. 300 µg was used as a reference value for all men and women older than 50 years but 400 µg was used as a reference value for women younger than 50 years.

**Table 5:** Baseline nutrient density of micronutrients compared to the recommended nutrient density given in NNR

	Men (*n*=46)	Women (*n*=100)		
			
Energy density of micronutrients	Mean (SD)	Mean (SD)	*P*	Recommended nutrient density^a^
Vitamin C (mg/MJ)^b^	9.3 (8.1)	13.0 (11.3)	0.001	8
Vitamin D (µg/MJ)^b^	0.6 (0.6)	0.6 (0.7)	0.768	1.0
Vitamin E (mg/MJ)	0.9 (0.3)	1.1 (0.4)	<0.001	0.9
Folate (µg/MJ)^b^	29.2 (14.7)	32.9 (12.4)	0.030	45
Calcium (mg/MJ)	114.7 (45.4)	110.0 (40.1)	0.528	100
Magnesium (mg/MJ)	40.0 (11.3)	41.5 (10.5)	0.443	35
Potassium (mg/MJ)	393.4 (112.4)	415.0 (118.9)	0.302	350
Sodium (mg/MJ)	353.9 (81.0)	330.9 (72.7)	0.090	–

Reference value not defined in the Nordic Nutrition Recommendations (NNR) (22).

*P* values refer to the difference in energy density of micronutrient intake between genders.

a Recommended nutrient density in NNR (22), to be used for planning diets for groups of individuals 6–60 years of age with heterogeneous age and sex distribution.

b Medians and interquartile range.

Only 20% of men and 26% of women met the RI of dietary fibre (25–35 g/day). More than 35 g/day of dietary fibre was consumed by 11% of men and 6% of women. When dietary fibre intake was energy adjusted (g of dietary fibre per MJ per day), 13% of men and 30% of women had recommended dietary fibre intake (gender difference, *P<*0.001) (Table 3). Sucrose intake was within the ≤10 E% limit in the diet of 80% of men and 74% of women. Higher intake of protein (E% and g/kg body weight) as well as alcohol (E% and g/d) was seen among men compared to women. The mean intake of alcohol among men was 3.5 E% (Table 3) and 35% of men and 24% of women in the current study consumed more than 5% of their total EI from alcohol.

Table 4 presents the average daily intake of micronutrients compared with RI, AR, and LI. Of the micronutrients studied, intake of vitamin D was most frequently below RI. Intake below RI was observed in 65% of men and 74% of women. Furthermore, 17% of men and 19% of women had intake below LI. No one exceeded the upper limit (50 µg) set for vitamin D intake. Folate intake below the RI was seen for 52% of men and 71% of women and 13% of men and 17% of women had intake below AR. Women of reproductive age are recommended an intake of 400 µg/day of folate (22). Women below the age of 50 (*n*=22) were considered to be of reproductive age (42) and only two women <50 years had folate intake ≥400 µg, corresponding to almost 91% of women of reproductive age not reaching the RI of folate.

Consumption of less than the estimated LI (400 mg) of calcium intake was seen among 5% of women. Sodium intake was on average higher than recommended and 83% of men and 65% of women were found to exceed the limits set for sodium intake. The sodium intake corresponded to a daily median intake of 8.8 g of salt for men and 6.7 g of salt for women.

Table 5 presents the vitamin and mineral intake as nutrient density. The intake of vitamin C, vitamin E, and folate per MJ was significantly higher among women than men. On average, the nutrient density was equal to or higher than recommended in the NNR for calcium, magnesium, potassium, vitamin C, and vitamin E. However, the nutrient density of vitamin D and folate was less than recommended for both genders.

## Discussion

The results of the present study of a Nordic adult population with MetS or at risk of developing MetS showed that the intake of SFA and sodium was higher and dietary fibre and PUFA intake was lower than recommended. In fact, >65% of the participants did not meet the recommendations (22) on SFA, PUFA, dietary fibre and sodium. About 20% of the participants had vitamin D intake below LI, possibly putting them at risk for developing diseases attributed to vitamin D deficiency symptoms (43). Another noteworthy result was the relatively high alcohol consumption observed among men and the gender difference in alcohol consumption seen amongst the participants in this cohort.

Vitamin D supplementation is commonly recommended in the Nordic countries due to lack of sun exposure (44, 45). Women planning on becoming pregnant are often recommended folate supplements (22, 45). Supplements were not included in the nutrient calculations and were supposed to be discontinued before the diet recording with the exception of vitamin D supplements in Iceland. Consequently, the total micronutrient intake might be somewhat underestimated in the population studied. In general, baseline nutrient intake in the SYSDIET study is in line with recent national surveys in the participating countries (45–48).

As the prevalence of MetS is increasing, the present study group is a highly relevant study group of adults in Westernised countries with an elevated risk of developing CVD and T2D. A balanced whole diet of adequate nutrient and food intake is of specific interest and importance in this risk group (49–51). The decision to develop joint NNR emerged not only from the geographical location of the Nordic countries but also from the similarities shared in dietary habits as well as in the prevalence of diet-related diseases, such as CVD, obesity, and T2D (3, 22).

Due to the design and aim of the study, all of the participants were overweight or obese, a large proportion had hypertension and elevated concentrations of plasma glucose. Furthermore, average lipid values were similar to previously reported results for people with MetS (52, 53). Some SYSDIET participants may have received dietary advice due to these elevated risk factors prior to the participation in the study. Thus, it is possible that this resulted in an underestimation of the poor adherence to dietary recommendations observed in this population. The present data may be useful in future Nordic prevention programmes of MetS, for example, identifying those nutrient targets that need special intervention.

This study revealed insufficient dietary fat quality in the study population. The contribution of SFA to total EI was above the RI level in about 80% of the participants. Replacement of SFA with PUFA in particular can improve the risk profile in the MetS (54) and lower the risk of CHD. In a meta-analysis by Mozaffarian et al. (49) researchers found 19% reduced risk of CHD events for intervention groups consuming on average 15% of total energy from PUFA, compared with the control groups with only 5% of total EI from PUFA. Thus, for each 5% increase in the proportion of energy obtained from PUFA when substituting SFA, the risk of CHD events was reduced by 10% (49). Higher intake of dietary fibre has been associated with lower risk for MetS, T2D, and CVD in high-risk populations (55–58). Two recent studies indicate that improved fat quality and fibre intake through the consumption of healthy Nordic food can play an important role in decreasing the risk of chronic disease (59, 60). More than half of the population studied was in treatment for hypertension. Given the known association between sodium (salt) intake and blood pressure, the high sodium intake in the present study is alarming. Lowering salt intake has been shown to decrease blood pressure in MetS subjects (61). Lower salt intake has also been shown to contribute to blood pressure reduction in hypertensive participants receiving medical therapy (62) and could decrease the risk of stroke and CHD (63).

Of the investigated vitamins and minerals, vitamin D intake was most frequently below RI for both genders with 65% of men and 74% of women not reaching the current RI of 7.5 µg/day. Nearly 20% of participants had an intake below LI for vitamin D. Vitamin D has gained a lot of attention with low levels of intake as well as low serum 25-hydroxyvitamin D being associated with several health risks besides low bone density traditionally related to vitamin D deficiency (64, 65). The fact that a considerable proportion of the participants had calcium intake below RI and 5% of the women had intake below LI, needs special attention in the population studied.

In the present study, the same inclusion criteria and the same method for dietary assessment were used in all six centres in the four Nordic countries. The need for comparable data on nutrient intake across Europe has been recognised as a task hampered by difficulties, complicated by the diverse study methodologies and varying purposes between studies (66). This multi-centre study carried out with comparable methodology is therefore an important step toward this goal. The present study gives novel and valuable information on the nutritional status of this cross-Nordic study group of people with MetS or MetS risk factors.

The NRV are primarily valid to assess intake on a group level. Comparison with reference values can only give some indication on whether the intake is adequate, but this does not mean that the requirements are met for each individual (22). Inherent errors related to the use of nutrient databases are unavoidable (17, 67). Accurate estimation of energy requirements using low-cost solutions like prediction equations are needed, but currently there is no consensus regarding this topic, especially when estimating the energy needs for obese individuals (68). Therefore, the limitations inherent with prediction equations such as HB apply here (68, 69). However, it is unlikely that application of other equations for the estimation of low-energy reporting would change our main findings and conclusions of considerably higher than recommended salt intake and low dietary fibre intake. The gender division in the current study is about one-third males and two-thirds females. In recent Finnish and Swedish population-based studies, the MetS prevalence was somewhat higher in men than women (9, 70). For better representativeness of our study population, a higher ratio of men would have been preferable. Higher numbers of participants and the addition of participants from the other Nordic countries would have given these results further value and generalisability to the remainder of the Nordic MetS population. However, there is no reason to assume that the nutritional intake of individuals with MetS would have been much different in other areas in the Nordic countries.

In conclusion, the dietary quality of this Nordic population with Mets or MetS risk factors is unsatisfactory and is characterised by high intakes of SFA and sodium and low intakes of PUFA and dietary fibre. Vitamin D intake estimated from the diet records was below RI level in a large part of the population. The low adherence to nutrition recommendations is likely to further perpetuate these high-risk individuals in developing T2D and CVD. Health providers should pay special attention to dietary assessments and should adequately educate these risk groups about the potential consequences of their nutritional intake toward the development of T2D and CVD. Authorities in the Nordic countries are encouraged to develop intervention programmes for high-risk groups. It seems relevant to implement programmes of guidelines and tests of their effects for people with Mets or MetS risk factors in the Nordic countries.

## References

[1] Astrup A (2001). Healthy lifestyles in Europe: prevention of obesity and type II diabetes by diet and physical activity. Public Health Nutr.

[2] Alberti KG, Eckel RH, Grundy SM, Zimmet PZ, Cleeman JI, Donato KA (2009). Harmonizing the metabolic syndrome: a joint interim statement of the International Diabetes Federation Task Force on Epidemiology and Prevention; National Heart, Lung, and Blood Institute; American Heart Association; World Heart Federation; International Atherosclerosis Society; and International Association for the Study of Obesity. Circulation.

[3] OECD http://ec.europa.eu/health/reports/docs/health_glance_2012_en.pdf.

[4] Aspelund T, Gudnason V, Magnusdottir BT, Andersen K, Sigurdsson G, Thorsson B (2010). Analysing the Large Decline in Coronary Heart Disease Mortality in the Icelandic Population Aged 25–74 between the Years 1981 and 2006. PLoS One.

[5] Hildrum B, Mykletun A, Hole T, Midthjell K, Dahl AA (2007). Age-specific prevalence of the metabolic syndrome defined by the International Diabetes Federation and the National Cholesterol Education Program: the Norwegian HUNT 2 study. BMC Public Health.

[6] Sundstrom J, Riserus U, Byberg L, Zethelius B, Lithell H, Lind L (2006). Clinical value of the metabolic syndrome for long term prediction of total and cardiovascular mortality: prospective, population based cohort study. BMJ.

[7] Miettola J, Nykanen I, Kumpusalo E (2012). Health views and metabolic syndrome in a Finnish rural community: a cross-sectional population study. Can J Rural Med.

[8] Stensvold D, Nauman J, Nilsen TIL, Wisloff U, Slordahl SA, Vatten L (2011). Even low level of physical activity is associated with reduced mortality among people with metabolic syndrome, a population based study (the HUNT 2 study, Norway). BMC Med.

[9] Pajunen P, Rissanen H, Harkanen T, Jula A, Reunanen A, Salomaa V (2010). The metabolic syndrome as a predictor of incident diabetes and cardiovascular events in the Health 2000 Study. Diabetes Metab.

[10] Ezzati M, Lopez AD, Rodgers A, Vander Hoorn S, Murray CJ (2002). Selected major risk factors and global and regional burden of disease. Lancet.

[11] World Health Organization 2009 2008–2013 action plan for the global strategy for the prevention and control of noncommunicable diseases.

[12] World Health Organization (WHO) Cardiovascular diseases (CVDs). Fact sheet N°317. http://www.who.int/mediacentre/factsheets/fs317/en/.

[13] Khaw KT, Wareham N, Bingham S, Welch A, Luben R, Day N (2008). Combined impact of health behaviours and mortality in men and women: the EPIC-Norfolk prospective population study. PLoS Med.

[14] World Health Organization (2003). Diet, nutrition, and the prevention of chronic diseases: report of a joint WHO/FAO expert consultation.

[15] Jonsdottir S, Hughes R, Thorsdottir I, Yngve A (2011). Consensus on the competencies required for public health nutrition workforce development in Europe – the JobNut project. Public Health Nutr.

[16] Jonsdottir S, Thorsdottir I, Kugelberg S, Yngve A, Kennedy NP, Hughes R (2012). Core functions for the public health nutrition workforce in Europe: a consensus study. Public Health Nutr.

[17] Slimani N, Deharveng G, Unwin I, Southgate DA, Vignat J, Skeie G (2007). The EPIC nutrient database project (ENDB): a first attempt to standardize nutrient databases across the 10 European countries participating in the EPIC study. Eur J Clin Nutr.

[18] Brussaard JH, Lowik MR, Steingrimsdottir L, Moller A, Kearney J, De Henauw S (2002). A European food consumption survey method – conclusions and recommendations. Eur J Clin Nutr.

[19] Ramel A, Martinez JA, Kiely M, Bandarra NM, Thorsdottir I (2010). Moderate consumption of fatty fish reduces diastolic blood pressure in overweight and obese European young adults during energy restriction. Nutrition.

[20] Kyro C, Skeie G, Dragsted LO, Christensen J, Overvad K, Hallmans G (2012). Intake of whole grain in Scandinavia: intake, sources and compliance with new national recommendations. Scand J Public Health.

[21] Zamora-Ros R, Knaze V, Lujan-Barroso L, Kuhnle GG, Mulligan AA, Touillaud M (2012). Dietary intakes and food sources of phytoestrogens in the European Prospective Investigation into Cancer and Nutrition (EPIC) 24-hour dietary recall cohort. Eur J Clin Nutr.

[22] (2004). Nordic Nutrition Recommendations 2004: integrating nutrition and physical activity.

[23] Uusitupa M, Hermansen K, Savolainen MJ, Schwab U, Kolehmainen M, Brader L (2013). Effects of an isocaloric healthy Nordic diet on insulin sensitivity, lipid profile and inflammation markers in metabolic syndrome – a randomized study (SYSDIET). J Intern Med.

[24] European Food Information Resource (EuroFIR) http://www.eurofir.eu.

[25] The Icelandic Food Composition Database (ISGEM) http://www.matis.is/ISGEM/en/.

[26] National Food Institute – Technical University of Denmark (DTU) (2009). Danish Food Composition Database – version 7.1. http://www.foodcomp.dk/v7/fcdb_default.asp.

[27] Livsmedelsverket – the National Food Agency Livsmedelsdatabasen. http://www.slv.se/en-gb.

[28] National Institute for Health and Welfare Fineli – Finnish Food Composition Database. http://www.fineli.fi.

[29] Black AE (2000). The sensitivity and specificity of the Goldberg cut-off for EI: BMR for identifying diet reports of poor validity. Eur J Clin Nutr.

[30] Goldberg GR, Black AE, Jebb SA, Cole TJ, Murgatroyd PR, Coward WA (1991). Critical-evaluation of energy-intake data using fundamental principles of energy physiology: 1. Derivation of cutoff limits to identify under-recording. Eur J Clin Nutr.

[31] Harris JA, Benedict FG (1918). A biometric study of human basal metabolism. Proc Natl Acad Sci USA.

[32] Hamwi GJ, Danowski TS (1964). Therapy: changing dietary concepts. Diabetes mellitus diagnosis and treatment.

[33] Salvino RM, Dechicco RS, Seidner DL (2004). Perioperative nutrition support: who and how. Cleve Clin J Med.

[34] Black AE, Coward WA, Cole TJ, Prentice AM (1996). Human energy expenditure in affluent societies: an analysis of 574 doubly-labelled water measurements. Eur J Clin Nutr.

[35] Black AE (2000). Critical evaluation of energy intake using the Goldberg cut-off for energy intake: basal metabolic rate. A practical guide to its calculation, use and limitations. Int J Obes Relat Metab Disord.

[36] Luhrmann PM, Herbert BM, Neuhauser-Berthold M (2001). Underreporting of energy intake in an elderly German population. Nutrition.

[37] Friedewald WT, Levy RI, Fredrickson DS (1972). Estimation of the concentration of low-density lipoprotein cholesterol in plasma, without use of the preparative ultracentrifuge. Clin Chem.

[38] Lankinen M, Schwab U, Kolehmainen M, Paananen J, Poutanen K, Mykkanen H (2011). Whole grain products, fish and bilberries alter glucose and lipid metabolism in a randomized, controlled trial: the Sysdimet study. PLoS One.

[39] Schwab U, Alfthan G, Aro A, Uusitupa M (2011). Long-term effect of betaine on risk factors associated with the metabolic syndrome in healthy subjects. Eur J Clin Nutr.

[40] Erkkila AT, Schwab US, de Mello VDF, Lappalainen T, Mussalo H, Lehto S (2008). Effects of fatty and lean fish intake on blood pressure in subjects with coronary heart disease using multiple medications. Eur J Nutr.

[41] Kolehmainen M, Salopuro T, Schwab US, Kekalainen J, Kallio P, Laaksonen DE (2008). Weight reduction modulates expression of genes involved in extracellular matrix and cell death: the GENOBIN study. Int J Obes (Lond).

[42] OECD Family Database (2012) OECD, Paris. http://www.oecd.org/social/family/database.

[43] Huotari A, Herzig KH (2008). Vitamin D and living in northern latitudes–an endemic risk area for vitamin D deficiency. Int J Circumpolar Health.

[44] Forsmo S, Fjeldbo SK, Langhammer A (2008). Childhood cod liver oil consumption and bone mineral density in a population-based cohort of peri- and postmenopausal women: the Nord-Trondelag Health Study. Am J Epidemiol.

[45] Thorgeirsdottir H, Valgeirsdottir H, Gunnarsdottir I, Gisladottir E, Gunnarsdottir BE, Thorsdottir I (2011). What do Icelanders eat? Dietary survey on the diet of Icelanders 2010–2011. Main results.

[46] Amcoff E, Edberg A, Barbieri HE, Lindroos AK, Nälsén C, Pearson M (2012). Riksmaten – vuxna 2010–11. Livsmedels-och näringsintag bland vuxna i Sverige.

[47] Helldan A, Kosonen M, Tapanainen H (2013). The National FINDIET 2012 Survey.

[48] Pedersen AN, Fagt S, Groth MV, Christensen T, Biltoft-Jensen A, Matthiessen J (2010). Dietary habits in Denmark 2003–2008. Main results.

[49] Mozaffarian D, Micha R, Wallace S (2010). Effects on coronary heart disease of increasing polyunsaturated fat in place of saturated fat: a systematic review and meta-analysis of randomized controlled trials. PLoS Med.

[50] Carlson JJ, Eisenmann JC, Norman GJ, Ortiz KA, Young PC (2011). Dietary fiber and nutrient density are inversely associated with the metabolic syndrome in US adolescents. J Am Diet Assoc.

[51] Aleixandre A, Miguel M (2008). Dietary fiber in the prevention and treatment of metabolic syndrome: a review. Crit Rev Food Sci Nutr.

[52] Pajunen P, Kotronen A, Korpi-Hyovalti E, Keinanen-Kiukaanniemi S, Oksa H, Niskanen L (2011). Metabolically healthy and unhealthy obesity phenotypes in the general population: the FIN-D2D Survey. BMC Public Health.

[53] Saaristo T, Moilanen L, Korpi-Hyovalti E, Vanhala M, Saltevo J, Niskanen L (2010). Lifestyle intervention for prevention of type 2 diabetes in primary health care: one-year follow-up of the Finnish National Diabetes Prevention Program (FIN-D2D). Diabetes Care.

[54] Bjermo H, Iggman D, Kullberg J, Dahlman I, Johansson L, Persson L (2012). Effects of n-6 PUFAs compared with SFAs on liver fat, lipoproteins, and inflammation in abdominal obesity: a randomized controlled trial. Am J Clin Nutr.

[55] Galisteo M, Duarte J, Zarzuelo A (2008). Effects of dietary fibers on disturbances clustered in the metabolic syndrome. J Nutr Biochem.

[56] McKeown NM, Meigs JB, Liu S, Saltzman E, Wilson PW, Jacques PF (2004). Carbohydrate nutrition, insulin resistance, and the prevalence of the metabolic syndrome in the Framingham Offspring Cohort. Diabetes Care.

[57] Sahyoun NR, Jacques PF, Zhang XLL, Juan WY, McKeown NM (2006). Whole-grain intake is inversely associated with the metabolic syndrome and mortality in older adults. Am J Clin Nutr.

[58] Weickert MO, Pfeiffer AF (2008). Metabolic effects of dietary fiber consumption and prevention of diabetes. J Nutr.

[59] Adamsson V, Reumark A, Fredriksson IB, Hammarstrom E, Vessby B, Johansson G (2011). Effects of a healthy Nordic diet on cardiovascular risk factors in hypercholesterolaemic subjects: a randomized controlled trial (NORDIET). J Intern Med.

[60] Olsen A, Egeberg R, Halkjaer J, Christensen J, Overvad K, Tjonneland A (2011). Healthy aspects of the Nordic diet are related to lower total mortality. J Nutr.

[61] Chen J, Gu D, Huang J, Rao DC, Jaquish CE, Hixson JE (2009). Metabolic syndrome and salt sensitivity of blood pressure in non-diabetic people in China: a dietary intervention study. Lancet.

[62] Frisoli TM, Schmieder RE, Grodzicki T, Messerli FH (2012). Salt and hypertension: is salt dietary reduction worth the effort?. Am J Med.

[63] Mozaffarian D, Appel LJ, Van Horn L (2011). Components of a cardioprotective diet: new insights. Circulation.

[64] Heaney RP (2008). Vitamin D in health and disease. Clin J Am Soc Nephrol.

[65] Holick MF, Chen TC (2008). Vitamin D deficiency: a worldwide problem with health consequences. Am J Clin Nutr.

[66] Vinas BR, Barba LR, Ngo J, Gurinovic M, Novakovic R, Cavelaars A (2011). Projected prevalence of inadequate nutrient intakes in Europe. Ann Nutr Metab.

[67] Poslusna K, Ruprich J, de Vries JHM, Jakubikova M, van't Veer P (2009). Misreporting of energy and micronutrient intake estimated by food records and 24 hour recalls, control and adjustment methods in practice. Brit J Nutr.

[68] Frankenfield D, Roth-Yousey L, Compher C (2005). Comparison of predictive equations for resting metabolic rate in healthy nonobese and obese adults: a systematic review. J Am Diet Assoc.

[69] Elizabeth Weekes C (2007). Controversies in the determination of energy requirements. Proc Nutr Soc.

[70] Novak M, Bjorck L, Welin L, Welin C, Manhem K, Rosengren A (2013). Gender differences in the prevalence of metabolic syndrome in 50-year-old Swedish men and women with hypertension born in 1953. J Hum Hypertens.

